# Conditional Use of Social and Private Information Guides House-Hunting Ants

**DOI:** 10.1371/journal.pone.0064668

**Published:** 2013-05-31

**Authors:** Adam L. Cronin

**Affiliations:** United Graduate School of Agricultural Sciences, Iwate University, Morioka, Iwate, Japan; Arizona State University, United States of America

## Abstract

Social animals can use both social and private information to guide decision making. While social information can be relatively economical to acquire, it can lead to maladaptive information cascades if attention to environmental cues is supplanted by unconditional copying. Ants frequently employ pheromone trails, a form of social information, to guide collective processes, and this can include consensus decisions made when choosing a place to live. In this study, I examine how house-hunting ants balance social and private information when these information sources conflict to different degrees. Social information, in the form of pre-established pheromone trails, strongly influenced the decision process in choices between equivalent nests, and lead to a reduced relocation time. When trails lead to non-preferred types of nest, however, social information had less influence when this preference was weak and no influence when the preference was strong. These results suggest that social information is vetted against private information during the house-hunting process in this species. Private information is favoured in cases of conflict and this may help insure colonies against costly wrong decisions.

## Introduction

Being well informed can mean the difference between a good and bad decision. Animals frequently make fitness-critical decisions based on information acquired through individual experience (private information) and via signals or cues from other animals (socially acquired information) [1]. Private information, while generally considered more reliable, can be costly or difficult to acquire [1], [2]. Social information, on the other hand, can be relatively cheap to obtain [3], [4] and, under the right circumstances, a more than adequate substitute [5], [6]. However, social information may also be outdated or unreliable, and a dependence on copying can lead to negative outcomes via information cascades [2], [7], [8]. Information cascades arise when individuals copy the behaviour of others without themselves assessing the environmental cues on which the behaviour was based, and can lead to sub-optimal outcomes when the individual copied chooses poorly [2], [9]. It is thus not surprising that many animals weight social or private information differently depending on the environmental context [10]–[14]. It remains unclear, however, in which context one form of information should be favoured over the other [7], [11].

Social insects exhibit some of the most sophisticated systems of information exchange, from the complex chemical messages encoded in chemical trails to the dance language of honey bees. These systems perform critical roles in the optimisation of collective processes [15]–[18]. Honey bees, for example, communicate the location of resources to other potential foragers via the waggle dance, varying the number of dance circuits with the quality of the resource [19]. Many other social insects coordinate foraging and colony movements using social information in the form of chemical (pheromone) trails [20]–[22]. Ants in particular rely on pheromone trails to recruit to food sources, and adaptively deploy these chemical signals based on the quality of the target [23], [24]. However, while both dance language and pheromone trails provide insect colonies with a means to effectively exploit available resources without the need for central control, there are notable differences in the flexibility of the two systems. Individuals interact directly to share information and recruitment is linear in honey bees, a dynamic communication system which enables colonies to switch sites rapidly in the case of resource depletion or new discoveries [15], [25]. Pheromone trails, on the other hand, are subject to momentum and runaway positive feedback because communication of information is indirect and recruitment is nonlinear [15], [25], [26]. As a result, once a trail is established, it may be difficult to switch targets [26]–[28]. This represents a form of information cascade [9] as although individuals continue to make independent assessments, the rapid amplification of initial choices (including poor ones) means that social information can soon overwhelm any dissent arising from private information, leading to potentially sub-optimal outcomes at the group level.

In addition to holding an important role in foraging, information exchange is critical to the process of finding a new home. Social insects relocate to a new nest when the present site becomes unsuitable or during the process of colony fission [29], and this process has been intensively studied in *Temnothorax* ants and honey bees [30], [31]. New sites are selected via a process of consensus decision making, in which a small proportion of the colony decide collectively from among candidate sites [32]. Scouts that have visited a suitable site share this information via waggle dances (honey bees) or by leading other scouts to the site via tandem running (*Temnothorax*) and, once a critical number of individuals (‘quorum’) is in favour of a particular site, the process shifts rapidly to that of relocation. A collective response emerges as a product of numerous individual decisions, each of which is made based on the private and social information available to that individual. In this manner, colonies are able to make accurate choices among sites of varying quality while maintaining colony integrity [31], [32].

Unlike other social insects so far studied (though see [33]), the small-colony ant *Myrmecina nipponica* relies on pheromone trails to navigate during house hunting [34]. Trails are laid by scouts that have found a suitable new nest site, leading to the recruitment of other nest-mate scouts and, once an apparently quorum-based threshold is reached, a switch to brood transport [34]. As in other species, scouts do not individually assess all candidate sites, and rely heavily on social information. However, as social information takes the form of a pheromone trail, consensus decisions made when selecting a new nest could be subject to information cascades. The initial sequence of events is decisive in this regard: if scouts first locate an acceptable, but sub-optimal nest and commence laying trails, subsequent scouts may be drawn to the same site over potentially superior sites. As pheromone trail strength increases, its influence over subsequent behaviour increases disproportionately [26], and the probability that scouts will locate other sites diminishes rapidly. Hence, if the initial sequence of events is sufficiently biased, an acceptable, but sub-optimal nest may eventually be selected. The cost of such wrong decisions during house hunting may be higher than during foraging, because all colony members (including queen and brood) are exposed during relocation and additional costs associated with the construction or modification of a new nest may be incurred. We might therefore expect private and social information to be weighted differently during house hunting. However, while studies have examined the use of conflicting social and private information in the context of ant foraging [35]–[38], and the possession of prior information is known to influence nest site selection [39], [40], no study to date has examined the effect of information conflict during house hunting (though see [41]). Furthermore, whereas information cascades are thought to explain a wide range of collective behaviours in humans, relatively little attention has been invested in the study of this concept in animal societies [27], [42], [43]. In this study, I examine how social and private information contribute to the consensus decision process during house-hunting in the ant *M. nipponica*, and assess whether the use of pheromone trails in this species can lead to information cascades during nest site selection.

## Methods

### Colony Collection and Maintenance

Entire colonies of *M. nipponica* (consisting of 14 to 65 ants) were collected from patches of moss and the bases of ferns in broadleaf forest on public land near Chitose City in Hokkaido, northern Japan (N42° 47′ E141° 34′, alt ∼100 m) in September 2011 and 2012. No permissions were required and this species is not protected. Colonies were housed in plastic boxes floored with plaster and containing an artificial nest consisting of a 2 mm high ring of foam covered with a microscope slide and red filter, and were kept in standard laboratory conditions (see [34] for more details).

### Colony Relocations

**Figure 1:**
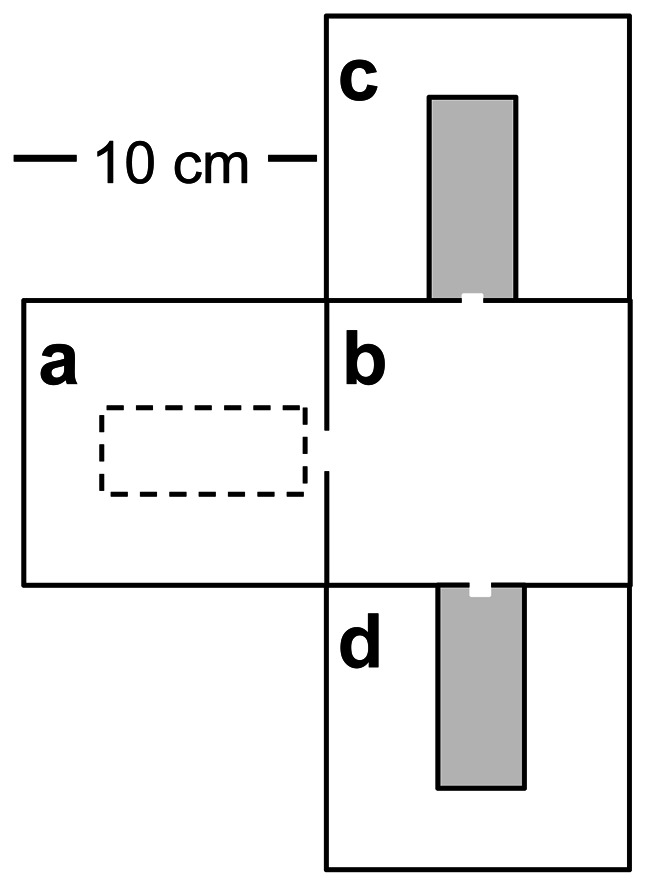
Modular experimental arena employing movable nest boxes. Individual boxes were 10×10 cm floored with 0.8 mm plaster and interconnected with small (10 mm) slots. New nests (grey shading) in destination boxes (c & d) were placed such that ants could only access the interior of the nest. Ant movements were thus restricted to the initial nest box (a) from which the original nest covering was removed (dotted line), the central navigation chamber (b), and the new candidate nests.

Colonies were forced to relocate between modular nest chambers (Figure 1) via ‘destruction’ of their home nest (see also [34]). This allowed the central navigation chamber, containing any chemical trails established during the relocation, to be rotated and swapped between trials (it is not possible to observe pheromone deposition in this species because the process is cryptic, and the manner in which it occurs (either passively or actively) is unknown). The possible influence of social information (pheromone trails) on nest site choice was investigated by forcing colonies to relocate via a navigation chamber with or without an established pheromone trail. An initial ‘lead’ colony was forced to relocate, thus establishing a pheromone trail in the navigation chamber (box b in Figure 1). These trails are not colony specific (see below). All boxes were then replaced except for the navigation chamber, and a second ‘follower’ colony was immediately forced to relocate. Unless otherwise stated, pheromone trails were established by a random laboratory colony not used in experiments. In all cases trails were established immediately prior to the running of experiments. Results indicated that the influence of pheromone trails became negligible after ∼24 hrs (see below), and all boxes were thoroughly scrubbed with water and sun-dried for at least 48 hrs prior to being reused.

### Pheromone Trail Longevity

To ensure that trails were effective at recruiting ants throughout experiments, the longevity of pheromone trail efficacy was assessed. A four-box choice arena was used (Figure 1) with a modified navigation chamber that restricted ant movement to narrow corridors (5 mm; ants are ∼3 mm long) connecting the source nest with two destination nests, forming a ‘T’ shape with the source nest at its base. An initial migration was carried out to establish a trail. Subsequently, the effect of this trail at various time intervals was tested as follows: individual ants were introduced to a chamber at the base of the ‘T’ and allowed to navigate to either the left of right branch of the ‘T’. These were then scored as selecting a branch if ants passed a half-way line along each branch. Ants that did not demonstrate a choice within two minutes were removed and classed as undecided. Ten ants from a single source colony that was different to the initial trail-laying colony were tested sequentially in this manner. A different test colony was used in each trial. Trial boxes were used for one test only and a total of 16 trials were run at times ranging from one to 26 hrs. A further ten control trials were run in a separate but identical arena in which no trail had been established. Only trials in which at least 8 of 10 ants tested chose a direction were included.

### Experiment 1: Influence of Social Information on Nest Choice

#### Efficacy of pheromone trails

To establish the colony specificity of pheromone trails and the efficacy of trails in influencing nest choice, six colonies were split into two separate groups, an ‘A’ group containing the queen, and a ‘B’ group, both of equal size and each assigned half of the colony’s brood. Each group was then forced to perform one relocation in each of three trials, with B groups taking the lead role and A groups following. In the first and second set of trials, both groups were from the same colony. In trial one the navigation chamber was left in the same orientation. In trial two the navigation box was rotated 90° after the lead group had relocated, to confirm that following groups were following trails and not just responding to the same environmental cues as lead groups (such as light or objects in the laboratory). In trial three, the navigation box was again rotated, and A groups were randomly assigned a B group from a different colony to test for colony specificity of pheromone trails.

#### Duration of relocation phases

To investigate how social information can influence the relocation process in more detail, six additional colonies of individually marked ants were forced to relocate using a three-box arrangement (i.e. with no nest choice, using boxes a-c in Figure 1). The process of nest relocation in *Myrmecina nipponica* is characterised by several stages, notably the discovery, assessment and transport phases [34]. Each colony performed two migrations which were randomly ordered; once with and once without pheromone trails, as for other experiments (see main text). The duration of the entire relocation, and of each phase (discovery phase: time from destruction of the nest to when the first ant enters the new nest; assessment phase: time from discovery until the first brood transport; and transport phase: time from first transport until end of relocation) was determined via video analysis of individual ant movements (see also [34]).

### Experiment 2: Effect of Maladaptive Social Information on Nest Choice

#### Preference testing

Colonies were first assessed for preferences between nests of varying traits to establish a baseline for selection trials. Colonies were provided with a choice between a normal nest (see above) or a treatment nest, with one of the following treatments: i) small nest: nest volume (length) was half of that or normal nests; ii) wide entrance: nest entrances were 10 mm instead of the usual 3 mm; iii) tall nest: nests were double height; iv) dry nest: nest boxes (plaster) were allowed to dry for 2 days prior to trials (whereas normal boxes were kept moist), v) dark versus light nests: one nest was covered with an opaque cover while the other was left with only a microscope slide (the red filter was removed). A group of 12 colonies was each tested once for each of the nest traits.

#### Effect of social information

The three most strongly preferred nest types (dark nest, wet nest, and narrow entrance; see below) were chosen for further trials. In the main experiment, an attempt was made to induce negative information cascades by priming colonies with an established pheromone trail leading to a non-preferred nest, in this way simulating an initial string of ‘bad choices’ by scouts. A paired experimental design was employed, with colonies performing one relocation with a trail and one without for each preference test. A total of 35 colonies were used in all trials and treatments were randomly ordered.

### Statistical Analysis

Results were analysed with the mixed-effects modelling (*lme*) procedure implemented in R version 2.15.2 [44]. Starting with all possible explanatory terms initially fitted, terms were removed in a stepwise fashion starting with interaction terms until the minimum adequate model was obtained as determined by comparison of values of Akaike Information Criterion [45]. Significance levels are reported for this minimum adequate model. In comparing relocation durations in experiment 1, a mixed-model was used with lead/follower status as fixed factor and colony as a random factor. For comparison of choices with and without trails in experiment 2, a binomial mixed-model was employed with treatment and trial type as fixed factors and colony as a random factor. Binomial linear models (*glm*) were used for analyses within trial groups as colonies were used only once each (for control and treatment) within trials. Colony size of lead and follower colonies had no influence in any of these tests. Exact binomial tests were used to test for preferences between nests of different traits (compared to a ‘no preference’ value of 0.5). Means are given as arithmetic mean ± standard deviation unless otherwise stated.

## Results

### Pheromone Trail Longevity

**Figure 2:**
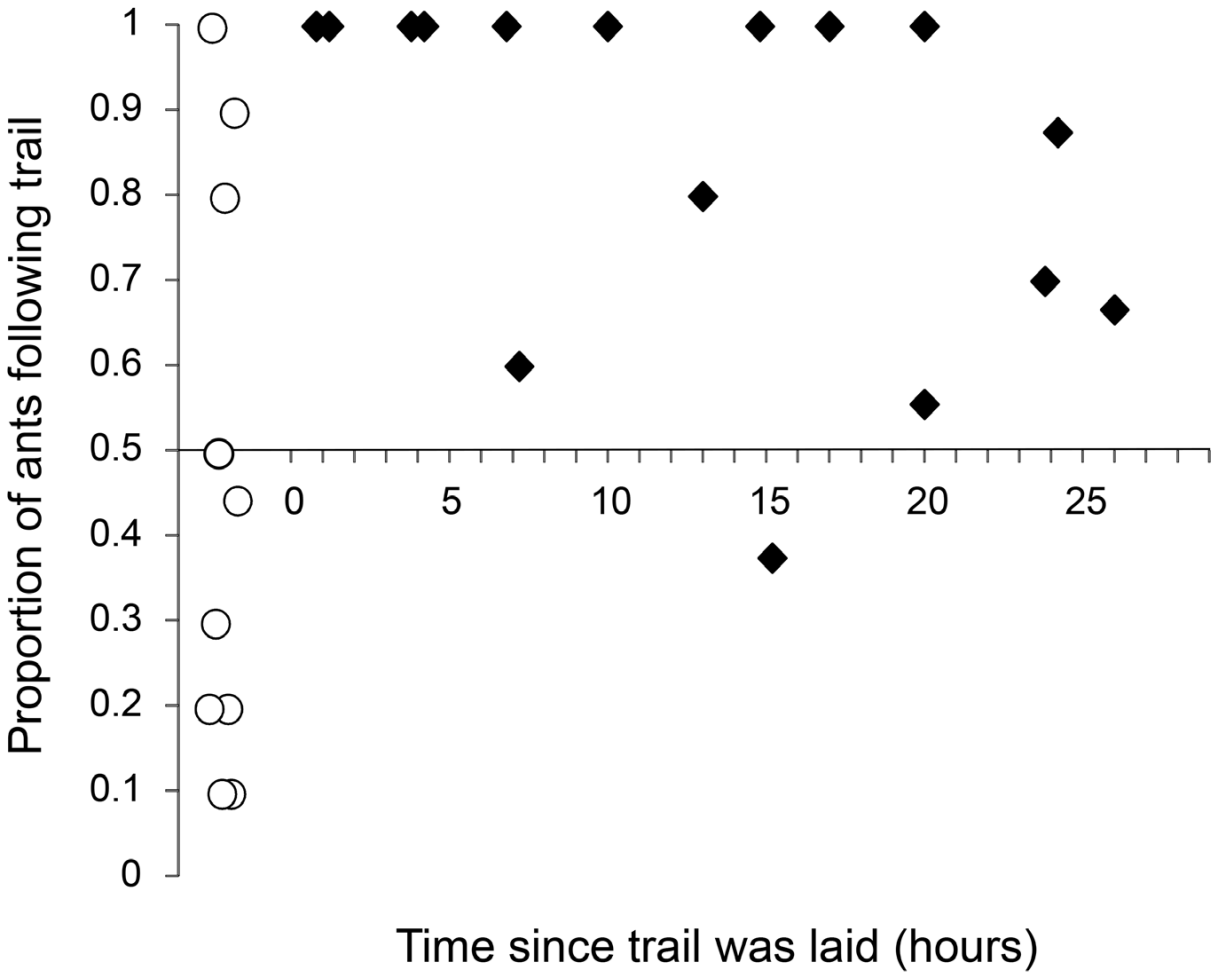
Duration of pheromone trail efficacy in *M.* *nipponica* in laboratory conditions. The x-axis represents time since the trail was laid (hours), while the y-axis represents the proportion of ants following the trail at time x. Ten ants were tested at each time period and given 2 minutes to select the left of right branch of a navigation chamber. Control trials (no trail) are indicated with white circles, whereas treatment trials (with trails) are indicated by black diamonds. Overlapping marks have been moved slightly for clarity.

Trails were followed by 100% of ants up to 4 hrs following trail establishment, and evidence suggests that trails were effective in influencing ant choice for ∼24 hrs (Figure 2). In control runs, and treatments in which trails were not followed by all ants, the order of choice in subsequent ants within a test group was largely random. For example, a typical test result was (L,R,R,L,R,R,x,R,L,L) where L is left, R is right and x is no choice. This suggests that individual test ants were not laying trails on the outgoing portion of the journey as has been demonstrated in some other species [23]. Hence repeated runs of individuals and colonies can be considered independent and the results an accurate reflection of trail duration, subject to differing environmental conditions in the lab. These data indicate that trails would have remained effective for the entire duration of relocation trials in the main experiment.

### Experiment 1: Influence of Social Information on Nest Choice

#### Efficacy of pheromone trails

Follower groups made the same choice as lead groups in all cases but one (*n* = 18; *Exact binomial test: p*<0.001), indicating that trails influence nest choice and that this effect is not colony-specific. Furthermore, follower groups relocated faster than lead groups, and this difference was significant when one outlier with a very long lead group time (colony 30B in Table 1) was removed from the analysis (*t*_4_ = 4.32, *p* = 0.0125; *n* = 16; mean for lead: 107±80 mins and follower: 64±32 mins; *n* = 18; Table 1).

**Table 1:** Influence of social information on choice between two identical nests.

Experiment	Lead group	Follower group	Lead choice	Follower choice	Lead duration	Follower duration
Same colony without rotation	21B	21A	N	N	Na	Na
	23B	23A	N	N	Na	Na
	26B	26A	N	N	Na	Na
	30B	30A	N	N	Na	Na
	52B	52A	S	S	Na	Na
	103B	103A	N	N	Na	Na
Same colony with rotation	21B	21A	N	S	1∶29	1∶07
	23B	23A	N	S	1∶03	0∶55
	**26B**	**26A**	**N**	**N**	**1∶36**	**2∶22**
	30B	30A	S	N	5∶15	0∶45
	52B	52A	N	S	1∶49	1∶02
	103B	103A	N	S	1∶43	1∶08
Foreign colony with rotation	21B	103A	S	N	1∶10	Na
	23B	52A	S	N	1∶15	0∶30
	26B	30A	N	S	1∶20	0∶50
	30B	26A	N	S	1∶10	1∶02
	52B	23A	N	S	1∶00	0∶41
	103B	21A	S	N	1∶45	Na

Colony groups were comprised of equally divided colonies (adults and brood) with queens in the ‘A’ colony. ‘Lead’ groups relocated followed by ‘follower’ groups, using the same navigation chamber (containing the pheromone trail). Navigation chambers were rotated 90° in some trials so that trails faced in the opposite direction for follower colonies. Choices are scored as ‘N’ or ‘S’ for north or south nest selected. Following the trail is indicated by the opposite choice in trials with rotation of the navigation chamber. The one instance where the ‘follower’ did not chose the same side as the ‘lead’ colony is shown in bold. ‘Na’ indicates data are not available. Follower relocations were of significantly shorter duration when the one outlier with a very long ‘lead’ time (colony 30 in “same colony with rotation” trials) was removed from the analysis (see main text). Times are given as hours:minutes.

#### Duration of relocation phases

Relocations undertaken using a three-box setup to examine phase durations concur with results above in that total relocation time was significantly shorter when colonies were provided with existing pheromone trails (mean with trail 62±13 minutes, without 98±27; *lme: t*_5_ = -3.23, *p* = 0.023; Table 2). Analysis of individual phases indicated that trails did not influence assessment time (*lme: t*_5_ = -1.87, *p* = 0.120) or transport time (*lme: t*_5_ = -1.26, *p* = 0.263), but that discovery time was significantly shorter with social information (*lme: t*_5_ = -3.24, *p* = 0.022). These data suggest that the reduction in overall relocation time in trials with trails is largely a result of a reduced time spent searching for a suitable site, though the same trend applied to both assessment and transport phases.

**Table 2:** Mean phase durations (mean ± SD in minutes) in relocating colonies with and without established pheromone trails.

	Discovery phase	Assessment phase	Transport phase	Total
With trail	2±3	40±21	21±5	62±13
No trail	14±8	57±27	27±11	98±27

Six individually marked colonies each performed one relocation with trails and one relocation without trails.

### Experiment 2: Effect of Maladaptive Social Information on Nest Choice

#### Preference testing

Colonies showed a significant preference for wet substrates over dry ones, and dark nests over light nests, but not between other traits tested (Table 3). There was however a non-significant trend toward selecting narrow entrances over wide entrances, a preference exhibited by other ant species [46]. These three traits were thus employed in subsequent trials, representing a ‘strong preference’ (for wet nests) and ‘weak preference’ (for narrow entrances and dark nests).

**Table 3:** Nest trait preference test results for relocating colonies of *M. nipponica*.

Nest trait	Trait preference		Statistic
Substrate	Wet 12	Dry 0	*p*<0.001
Nest	Dark 10	Light 2	*p = *0.039
Entrance	Narrow 9	Wide 3	*p* = 0.146
Nest	Big 7	Small 5	*p* = 0.774
Nest	Short 7	Tall 5	*p* = 0.774

Number of nests of each trait chosen in each of 12 trials indicated for each test group. Different colonies were used in each of the 12 tests in each group. Significance values are given for exact binomial tests when compared to an expected value of 50%.

#### Effect of social information

**Figure 3:**
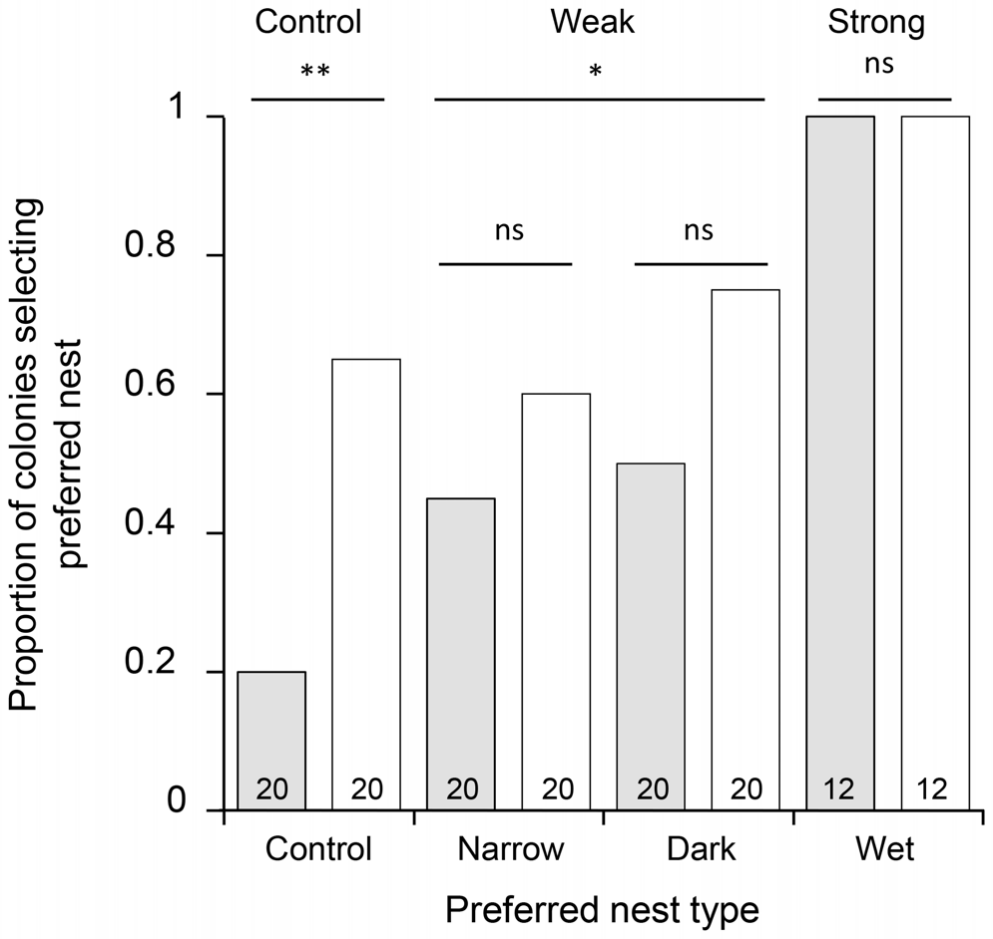
Proportion of colonies selecting preferred nests with and without pre-established pheromone trails. White bars indicate trials conducted without trails whereas grey bars indicate the presence of established trails. When present, trails always led to non-preferred nests. In control tests the ‘non-preferred’ nest was randomly allocated. The preferred nest in each trial set is indicated on the x-axis (see main text). Significance values for the effect of trails within each treatment group and for pooled data from weak preference trials are indicated above horizontal bars (* <0.05; ** <0.01; see also main text). The number of trials is in each case given at the bottom of the bar.

Results of choice tests without trails aligned well with initial preference trials, indicating a strong preference for wet over dry nests, and a weaker preference for dark over light nests (Figure 3). As for experiment 1, trail presence influenced choice between equivalent nests (*glm* for control: *z* = 2.749, *p* = 0.006). A similar trend was observed for colonies in weak preference trials, which selected a higher proportion of non-preferred nests when provided with trails (Figure 3). While this effect was not significant within groups (*glm* for dark/light: *z = *1.608, *p = *0.108; and narrow/wide: *z = *0.946, *p = *0.344), it was marginally significant when data from both weak preference trials were pooled (*lme: z = *2.034, *p* = 0.042). The effect of trails was also significant over all trials (*lme*: *z* = 3.203, *p* = 0.0014), though in contrast to other treatments, colonies in strong preference trials selected the preferred nest regardless of trail presence. Both nests were inspected by ants in all trials and no colony splitting was observed during relocations.

## Discussion

House-hunting *M. nipponica* colonies were strongly influenced by existing pheromone trails in choices between equivalent nests, and able to exploit this social information to reduce relocation time. In contrast, trails had no influence on choice when they lead to strongly non-preferred (dry) nests, while in weak preference trials, a higher proportion of colonies again selected non-preferred nests in the presence of trails. The selection of a new nest site is an emergent consequence of numerous semi-independent individual choices based on social and private information. In the main experiment social information (as represented by a fully established trail) was constant, whereas private information obtained by ants visiting non-preferred nests varied in the degree to which it conflicted with social information, from nil (in controls) to high (in strong preference trials). House hunting ants and bees visiting a given site assess its quality and either decide to accept it and begin recruiting to the site, or otherwise continue searching [31], [47]–[49]. This decision is independent for each individual in that scouts assess the site for themselves, but dependent on other colony members as scouts are more likely to visit sites already flagged by social information [8]. In this study, when private and social information were not in conflict, social information in the form of established trails influenced nest site selection. Trails recruited ants to the site and, on finding the site suitable, these ants presumably reinforced the trail, eventually giving rise to a consensus response. In circumstances where social information conflicted with private information, however, social information was less effective in influencing the colony level response. In strong preference trials, ants eschewing highly non-preferred (dry) sites were able to establish a competitive trail to the alternative site, eventually leading to an optimal colony level response in all twelve trials despite the established trail. In weak preference trials, a higher proportion of colonies selected the non-preferred nest in the presence of trails, though this effect was somewhat muted relative to that observed in controls. This suggests that social information was in some cases sufficient to bias nest site selection despite contradictory private information, presumably because weakly non-preferred nests (light nests and wide entrance nests) had sufficient support among scouts to give rise to a consensus response before a suitably competitive trail could be developed to the alternative site. That is, in weak preference trials the influence of pre-established social information was sufficient to overcome the low level of dissent arising from private information and influence nest choice. This represents a form of information cascade, as although copying is not unconditional, a sub-optimal group level outcome can arise via positive feedback following initial poor choices (represented here by the established trail). Information cascades are thought to explain a range of rapid, broad-scale emulative responses observed in humans [2], [9], and the available evidence suggests this may also be the case in animal societies [27], [28], [42], [43].

The relative cost and reliability of private and social information is thought to regulate the conditional weighting of one over the other [1], [3], [4]. The ‘costly information hypothesis’ suggests that social information should be preferred when private information is difficult to obtain [3], [4], and is supported by empirical studies in vertebrates [50], [51]. In house-hunting *Myrmecina*, the cost of acquiring private information is almost certainly high: scouts move slowly and thus finding a new home takes time, during which scouts risk desiccation, predation, and getting lost, and the colony as a whole is exposed. In addition, at least two other factors suggest social information should be favoured in this context. Firstly, social information in the form of pheromone trails can be considered reliable because it is provided by nest-mates with shared interests and the ephemeral nature of trails means that information is up to date. Secondly, Rendell *et al*. [5], [11] suggest that copying should be adaptive provided that the individuals which are copied behave rationally and select the best option. Ants deploy pheromone trails in proportion to the quality of the target both in foraging [24], [26] and in nest site selection [52], [53], and thus the very existence of a trail implies that an adaptive choice has been made. These arguments suggest that social information should be highly valued in relocating *M. nipponica* and, while this supposition is perhaps reflected in the strong influence of trails on choices between equivalent nests, the reduced influence of trails leading to non-preferred nests suggests that social information is not blindly accepted, but vetted against private information before a decision is made. These data support previous studies indicating that in the event of conflict ants defer to private information [36], [38], [54], [55]. Stroeymeyt *et al.* [41] showed that house hunting *Temnothorax* ants relied on prior experience (navigational memory) when chemical markings in their laboratory arena were experimentally reversed. This suggests a preference for private information over social information, though ants of this genus rely on tandem-running for recruitment during consensus decision making [48] and the role of chemical markings differs to that in *Myrmecina* [33]. Maintenance of some degree of independence in collective decisions such as this is perhaps not surprising as an individual component to choice is thought to be integral to an effective quorum response [32], and this vetting process may also buffer colonies against negative information cascades [8]. Studies of ants have also revealed the use of negative feedback mechanisms which can curb potential runaway positive feedback associated with the use of pheromone trails [56]–[58]. Vetting and damping systems such as these may be common in species that employ feedback mechanisms subject to information cascades, particularly when negative outcomes have potentially high fitness consequences.

Conditional use of information sources appears to be common in animal learning, and present evidence suggests it is probably widespread both taxonomically (reviews for fish [12], mammals [59], and birds [60]) and in terms of the context in which is it employed [7], [12]. Ants have been shown to use social and private information conditionally when foraging [10], [35], [61] and, combined with the present study, this suggests that at least in ants (i) the use of social and private information is not mutually exclusive and (ii) social information is not blindly accepted but vetted against private information, with deference to the latter in circumstances of conflict. While data presented here can be interpreted to largely support the costly information hypothesis, they suggest that even in situations where copying occurs, asocial learning is maintained and may function as an insurance mechanism against negative information cascades. The extent to which similar mechanisms can be found in other species is at present unknown, and indeed, there is a paucity of empirical experiments investigating the potential for maladaptive decisions arising from the use of social information [7]. Rieucau and Giraldeau [42] demonstrated that nutmeg manikins (*Lonchura punctulata*) could be induced to make maladaptive decisions when strong social information apparently lead them to disregarded personal information in a manner consistent with information cascades. Maladaptive choices connected with the use of social information have also been implicated in fish [62] and young birds [63]. Nonetheless, while information cascades are purported to explain a diverse range of human collective responses such as consumer fads and crowd panic behaviour [9] and have the support of laboratory studies [64], surprisingly few corresponding investigations have been undertaken in other animals. The growing body of literature on the subject demonstrates the importance of social information in the adaptive behaviour of animals, and future studies should investigate the potential for information cascades to influence conditional information use in other species.
